# Supplementary material for: Assessing the Anthelmintic Candidates BLK127 and HBK4 for Their Efficacy on Haemonchus contortus Adults and Eggs, and Their Hepatotoxicity and Biotransformation
Source: Pharmaceutics. 2022 Mar 30;14(4):754. doi: 10.3390/pharmaceutics14040754 (PMC9024958; doi:10.3390/pharmaceutics14040754)
Supplement: Supplementary file 1 [file pharmaceutics-14-00754-s001.zip › pharmaceutics-1649146-supplementary/Figure S1_UHPLC chromatograms and HRMS-MS spectrum of BLK127.pdf]

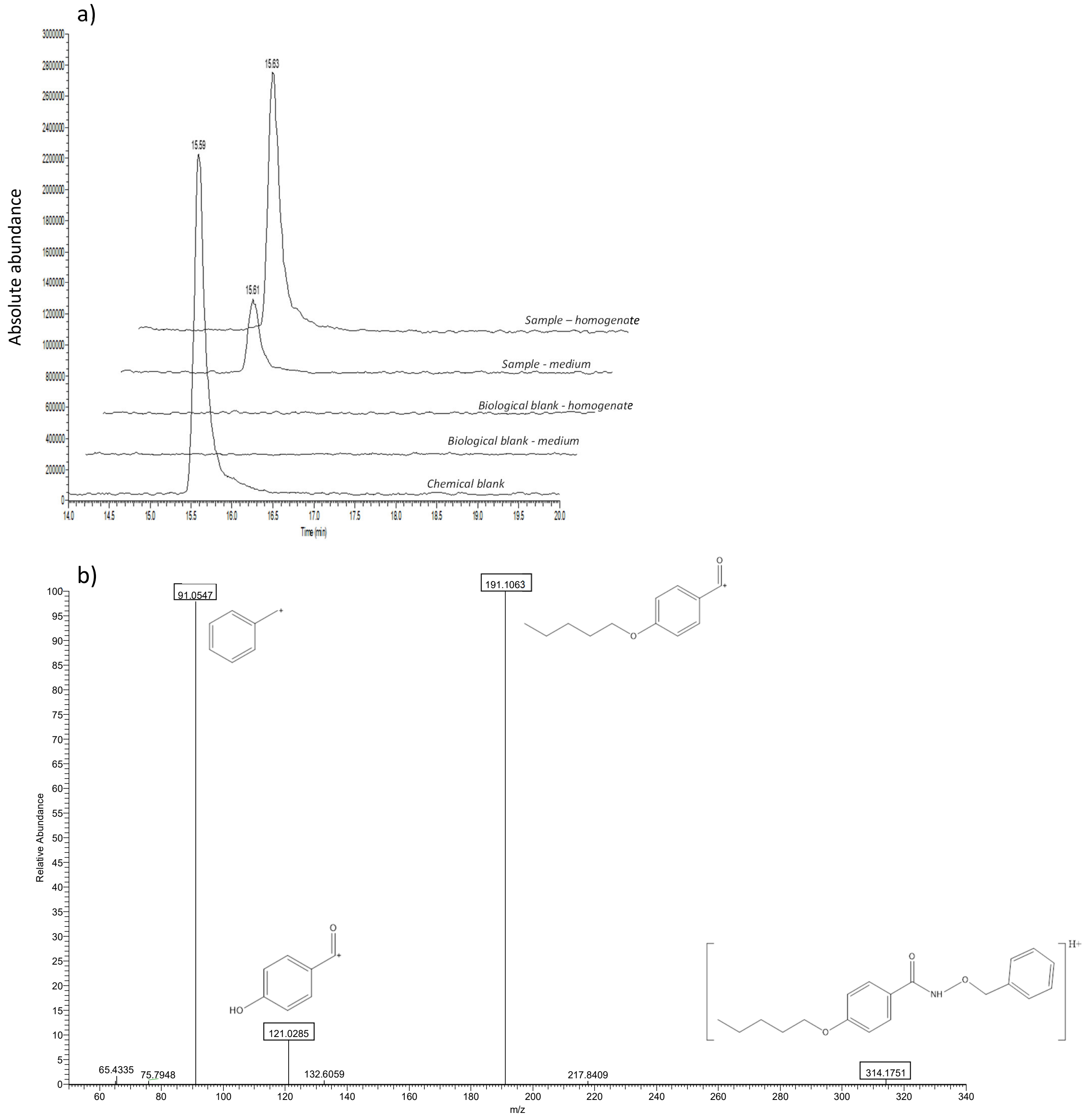

Figure S1: UHPLC chromatograms and HRMS/MS spectrum of BLK127. (a) Comparison of extracted ion UHPLC-HRMS chromatograms for *m/z* 314.1571 [M-H]$^+$ (BLK127) from *H. contortus* (IRE female) incubated with BLK127 (*sample–homogenate* and *sample–medium*), blank biological samples (*biological blank–homogenate* and *biological blank–medium*) and *chemical blank*. **(b)** HRMS/MS spectrum of BLK127 from sample–homogenate with proposed structures of the main fragments.
