# Supplementary material for: Assessing the Anthelmintic Candidates BLK127 and HBK4 for Their Efficacy on Haemonchus contortus Adults and Eggs, and Their Hepatotoxicity and Biotransformation
Source: Pharmaceutics. 2022 Mar 30;14(4):754. doi: 10.3390/pharmaceutics14040754 (PMC9024958; doi:10.3390/pharmaceutics14040754)
Supplement: Supplementary file 1 [file pharmaceutics-14-00754-s001.zip › pharmaceutics-1649146-supplementary/Figure S2_UHPLC chromatograms and HRMS-MS spectra of BLK127-N-glc.pdf]

a)

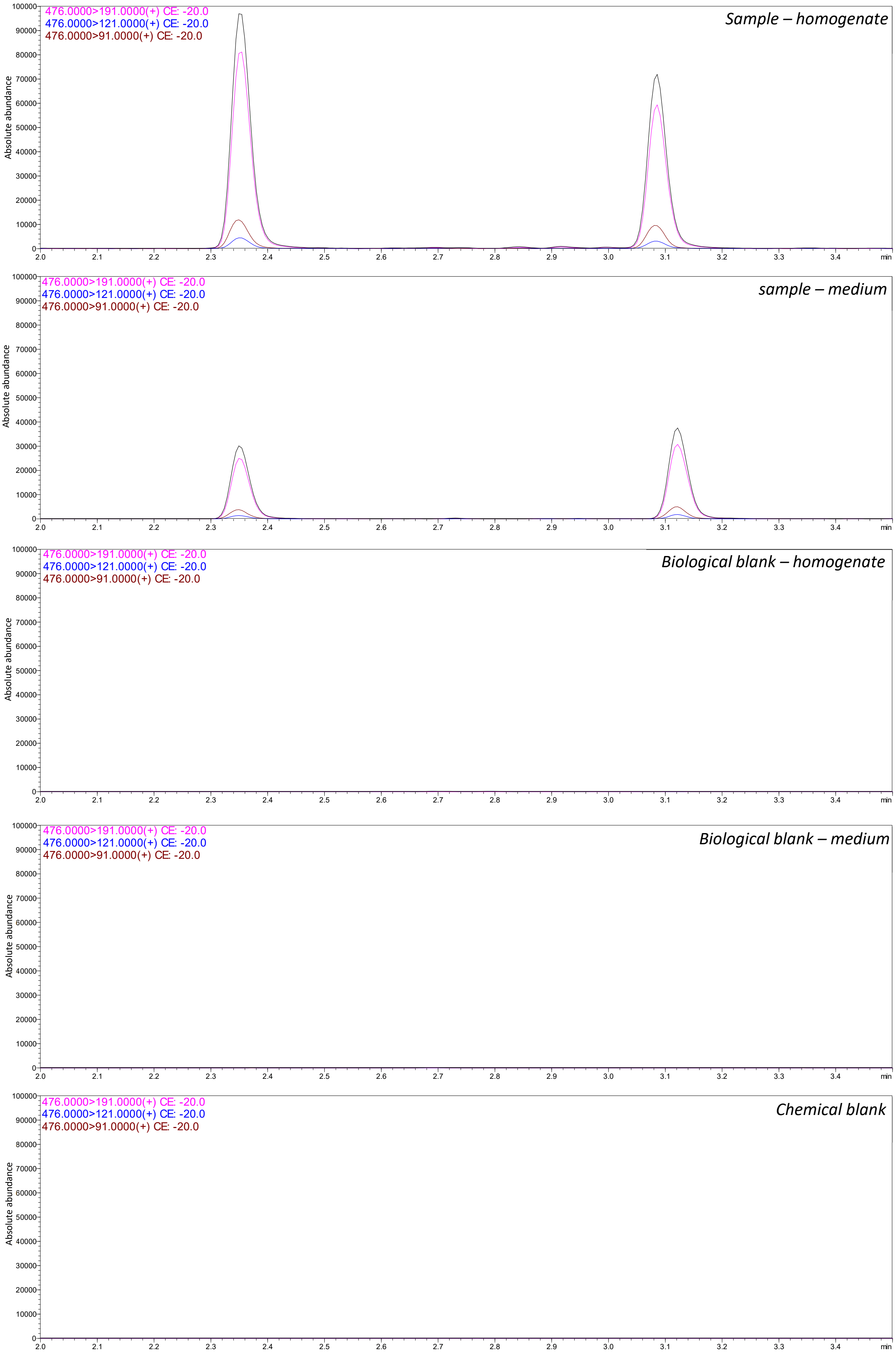
476.0000>191.0000(+) CE: -20.0
476.0000>121.0000(+) CE: -20.0
476.0000>91.0000(+) CE: -20.0
Sample – homogenate
sample – medium
Biological blank – homogenate
Biological blank – medium
Chemical blank
Absolute abundance
min

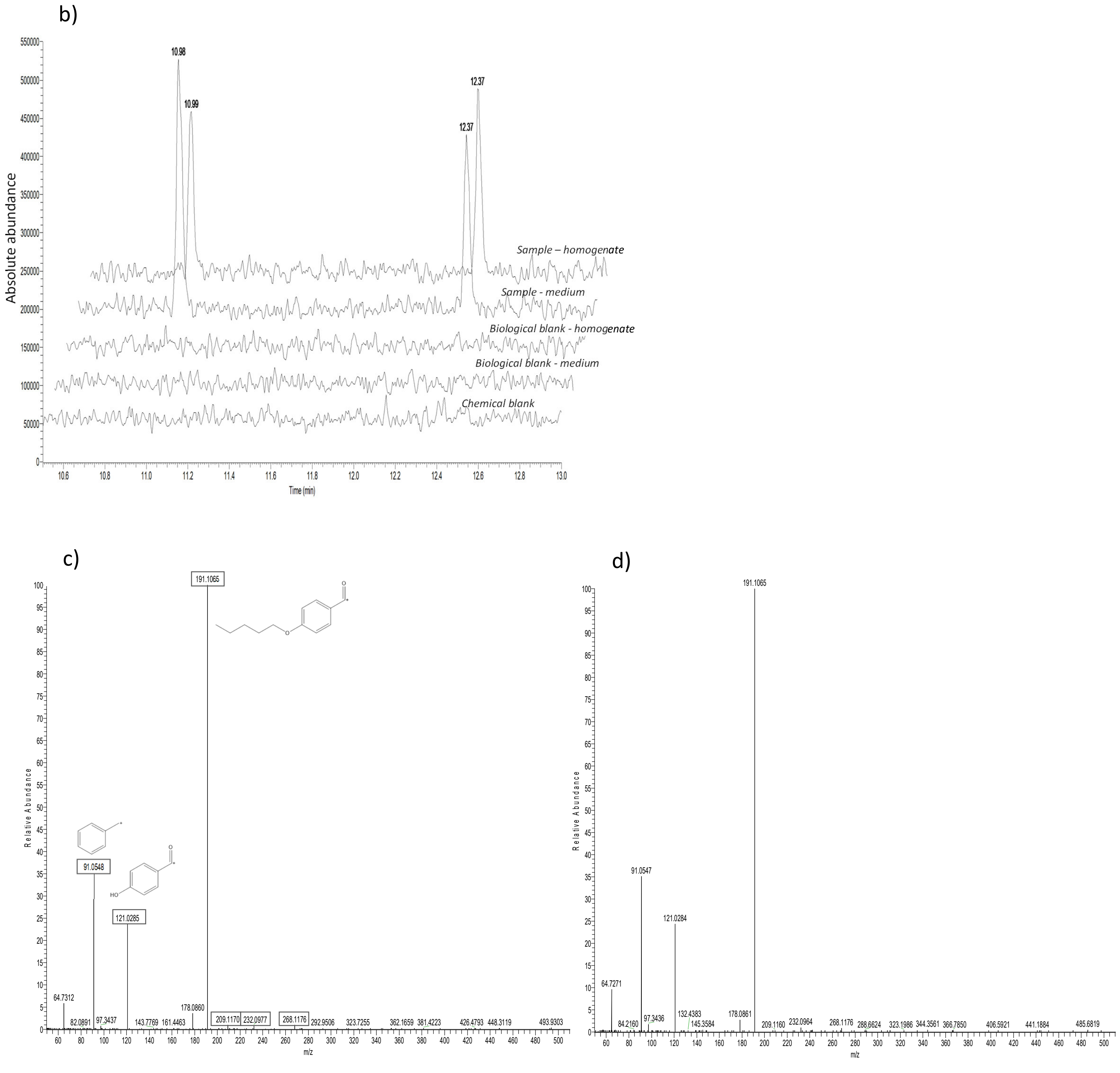

Figure S2: UHPLC chromatograms and HRMS/MS spectra of BLK127-N-glc (a) Representative UHPLC-MS/MS chromatograms of BLK127-N-glc with MRM* in the samples incubated with BLK127 (*sample–homogenate* and *sample–medium*), blank biological samples (*biological blank–homogenate* and *biological blank–medium*) and *chemical blank*. **(b)** Comparison of extracted ion UHPLC-HRMS chromatograms for *m/z* 476.2279 $[M-H]^+$ (BLK127-N-glc) from *H. contortus* (IRE strain female) in the samples and blank samples. **(c)** HRMS/MS spectrum of BLK127-N-glc in t_R 10.99 min with the proposed structures of the main fragments. **(d)** HRMS/MS spectrum of BLK127-N-glc in t_R 12.37 min.

* multiple reaction monitoring
