# Supplementary material for: Assessing the Anthelmintic Candidates BLK127 and HBK4 for Their Efficacy on Haemonchus contortus Adults and Eggs, and Their Hepatotoxicity and Biotransformation
Source: Pharmaceutics. 2022 Mar 30;14(4):754. doi: 10.3390/pharmaceutics14040754 (PMC9024958; doi:10.3390/pharmaceutics14040754)
Supplement: Supplementary file 1 [file pharmaceutics-14-00754-s001.zip › pharmaceutics-1649146-supplementary/Figure S3_UHPLC chromatograms and HRMS-MS spectrum of h-BLK127.pdf]

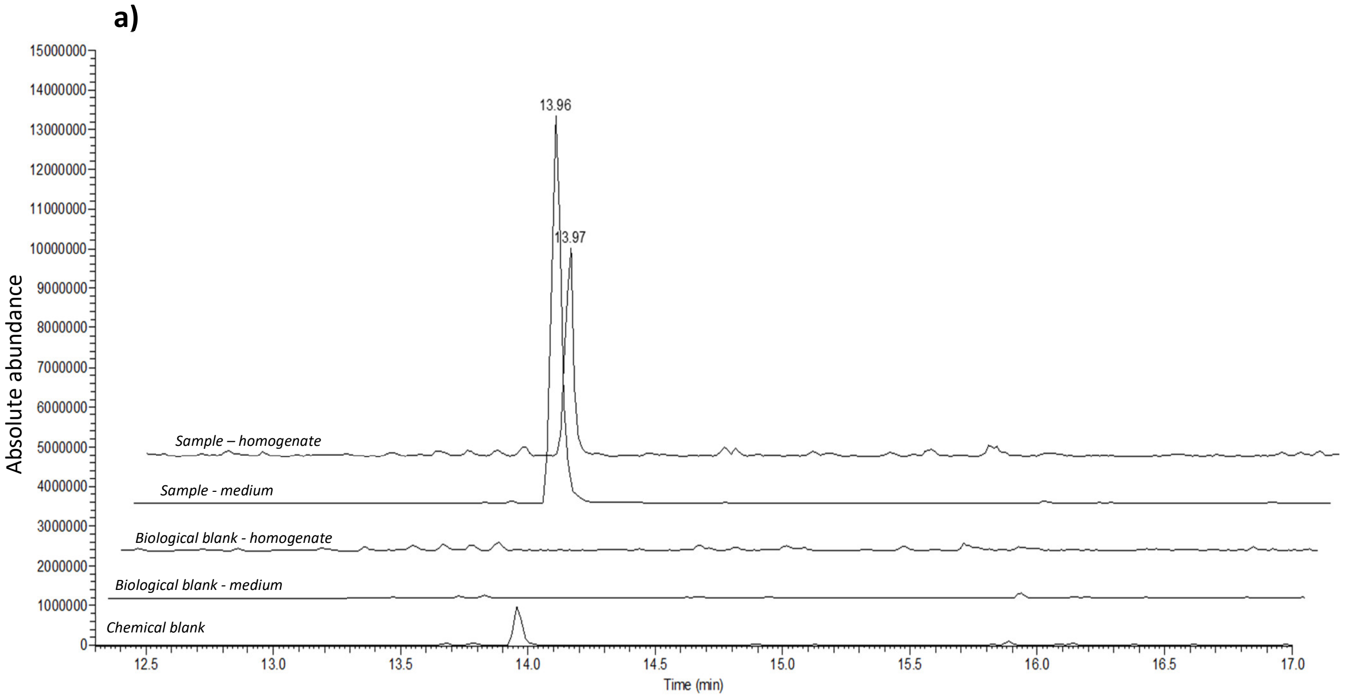
a)
Absolute abundance
15000000
14000000
13000000
12000000
11000000
10000000
9000000
8000000
7000000
6000000
5000000
4000000
3000000
2000000
1000000
0
13.96
13.97
Sample – homogenate
Sample - medium
Biological blank - homogenate
Biological blank - medium
Chemical blank
12.5
13.0
13.5
14.0
14.5
15.0
15.5
16.0
16.5
17.0
Time (min)

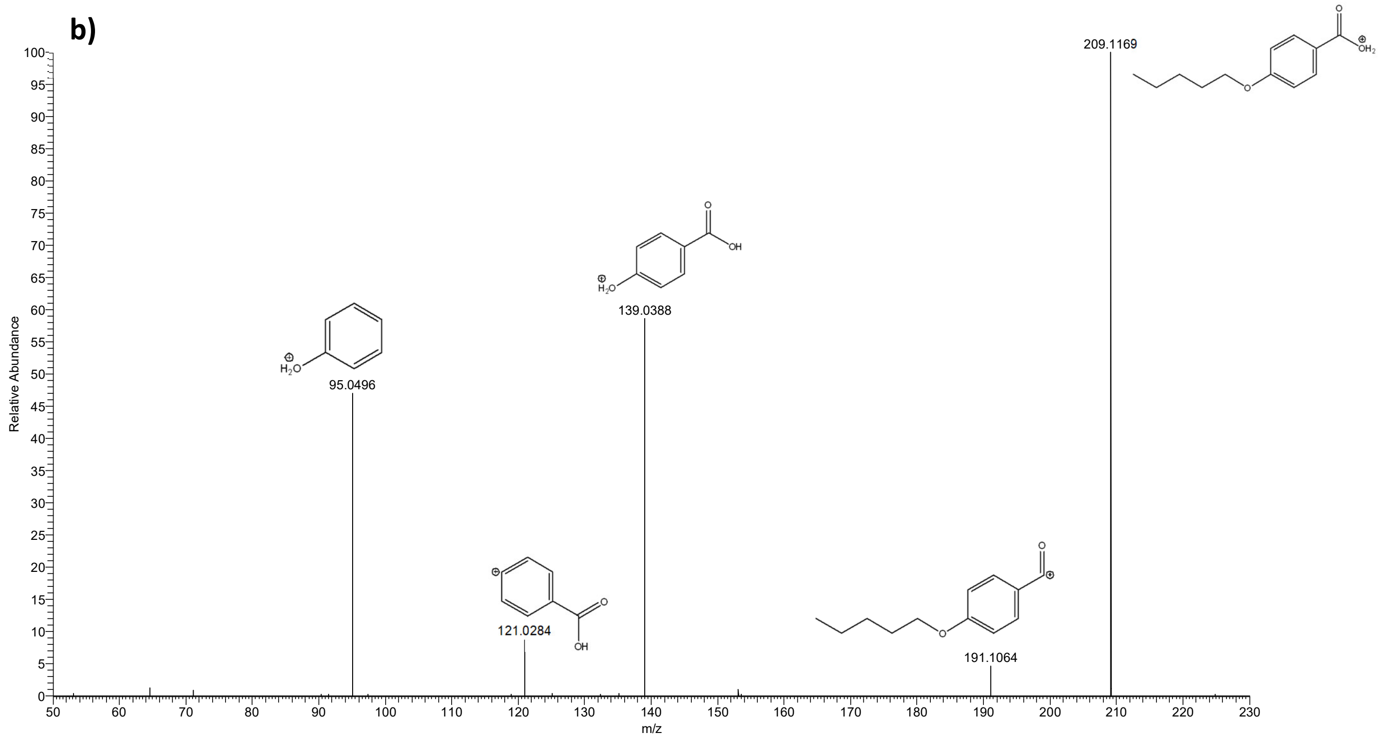
b)
Relative Abundance
209.1169
139.0388
95.0496
121.0284
191.1064
m/z
50
60
70
80
90
100
110
120
130
140
150
160
170
180
190
200
210
220
230

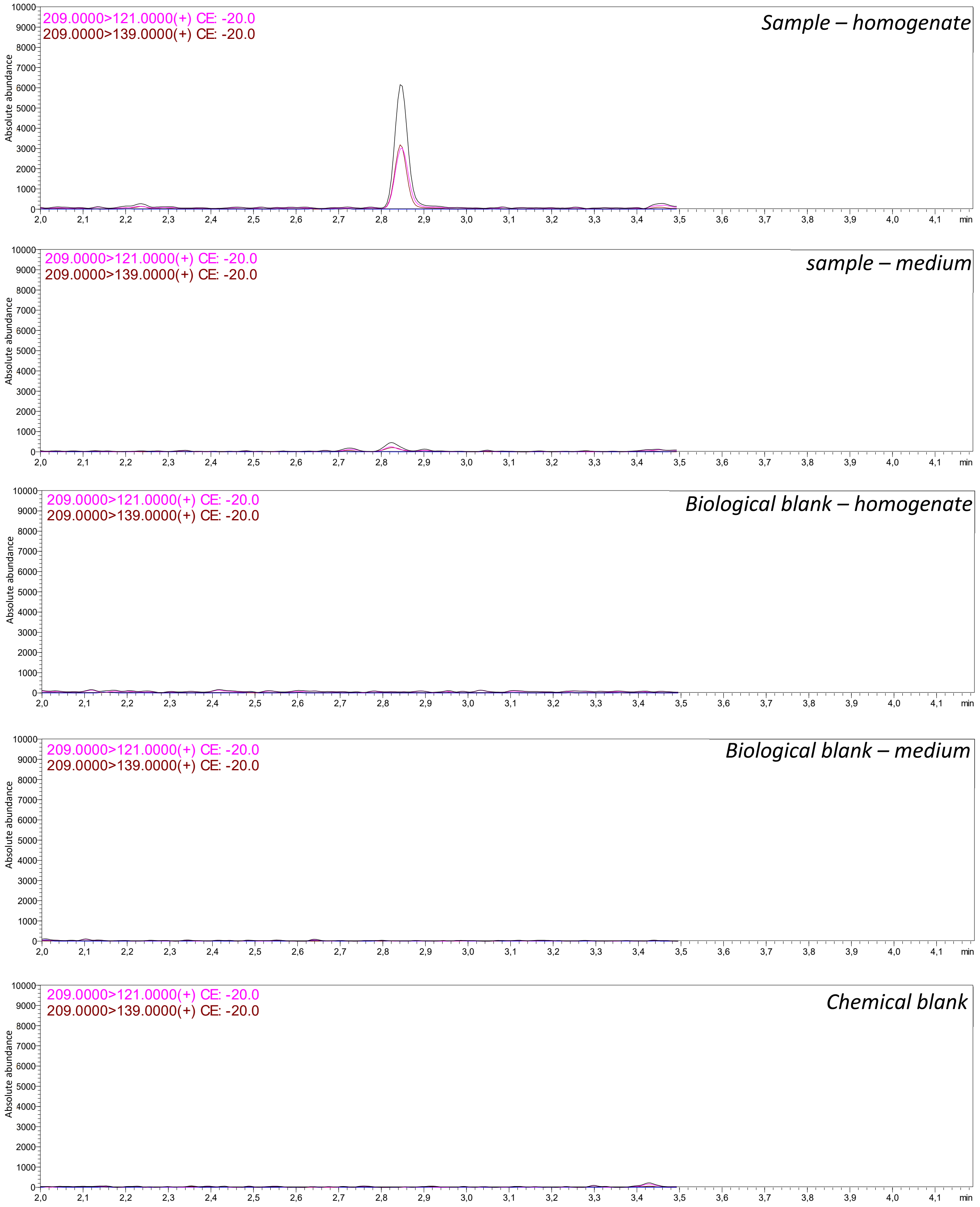

Figure S3: UHPLC chromatograms and HRMS/MS spectrum of h-BLK127. (a) Comparison of extracted ion UHPLC-HRMS chromatograms for *m/z* 209.1169 $[M-H]^{+}$ (h-BLK127) from *H. contortus* (IRE female) incubated with BLK127 (*sample–homogenate* and *sample–medium*), blank biological samples (*biological blank–homogenate* and *biological blank–medium*) and *chemical blank*. **(b)** HRMS/MS spectrum of h-BLK127 from *sample–homogenate* with proposed structures of the main fragments. **(c)** Representative UHPLC-MS/MS chromatograms of h-BLK127 in the samples and blank samples with MRM*.

* multiple reaction monitoring
