# Supplementary material for: Assessing the Anthelmintic Candidates BLK127 and HBK4 for Their Efficacy on Haemonchus contortus Adults and Eggs, and Their Hepatotoxicity and Biotransformation
Source: Pharmaceutics. 2022 Mar 30;14(4):754. doi: 10.3390/pharmaceutics14040754 (PMC9024958; doi:10.3390/pharmaceutics14040754)
Supplement: Supplementary file 1 [file pharmaceutics-14-00754-s001.zip › pharmaceutics-1649146-supplementary/Figure S5_ UHPLC chromatograms and comparison of HRMS spectra of BLK127-O-glc.pdf]

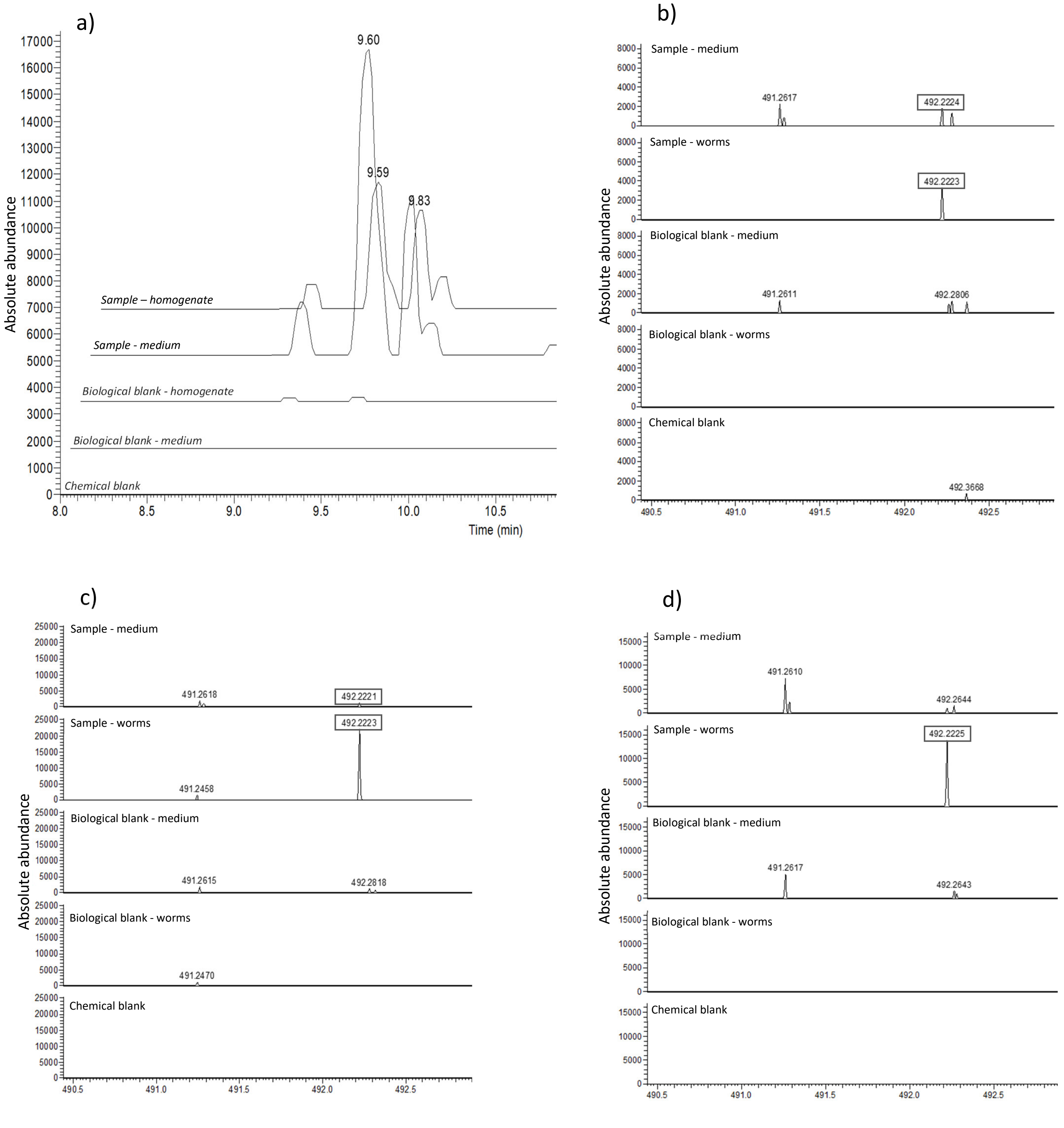

Figure S1: UHPLC chromatograms and comparison of HRMS spectra in the samples and blank samples of BLK127-O-glc. **(a)** Comparison of extracted ion UHPLC-HRMS chromatograms for *m/z* 492.2224 $[M-H]^+$ (BLK127-O-glc) from *H. contortus* (IRE strain, female) incubated with BLK127 (*sample–homogenate* and *sample–medium*), blank biological samples (*biological blank–worms* and *biological blank–medium*) and *chemical blank.* **(b)** Comparison of full HRMS spectra in the samples and blank samples in t_R 9.20 min. **(c)** Comparison of full HRMS spectra in the samples and blank samples in t_R 9.60 min. **(d)** Comparison of full HRMS spectra in the samples and blank samples in t_R 9.83 min.
