# Supplementary material for: Assessing the Anthelmintic Candidates BLK127 and HBK4 for Their Efficacy on Haemonchus contortus Adults and Eggs, and Their Hepatotoxicity and Biotransformation
Source: Pharmaceutics. 2022 Mar 30;14(4):754. doi: 10.3390/pharmaceutics14040754 (PMC9024958; doi:10.3390/pharmaceutics14040754)
Supplement: Supplementary file 1 [file pharmaceutics-14-00754-s001.zip › pharmaceutics-1649146-supplementary/Figure S6_UHPLC chromatograms and HRMS-MS spectrum of h-BLK127-gly. .pdf]

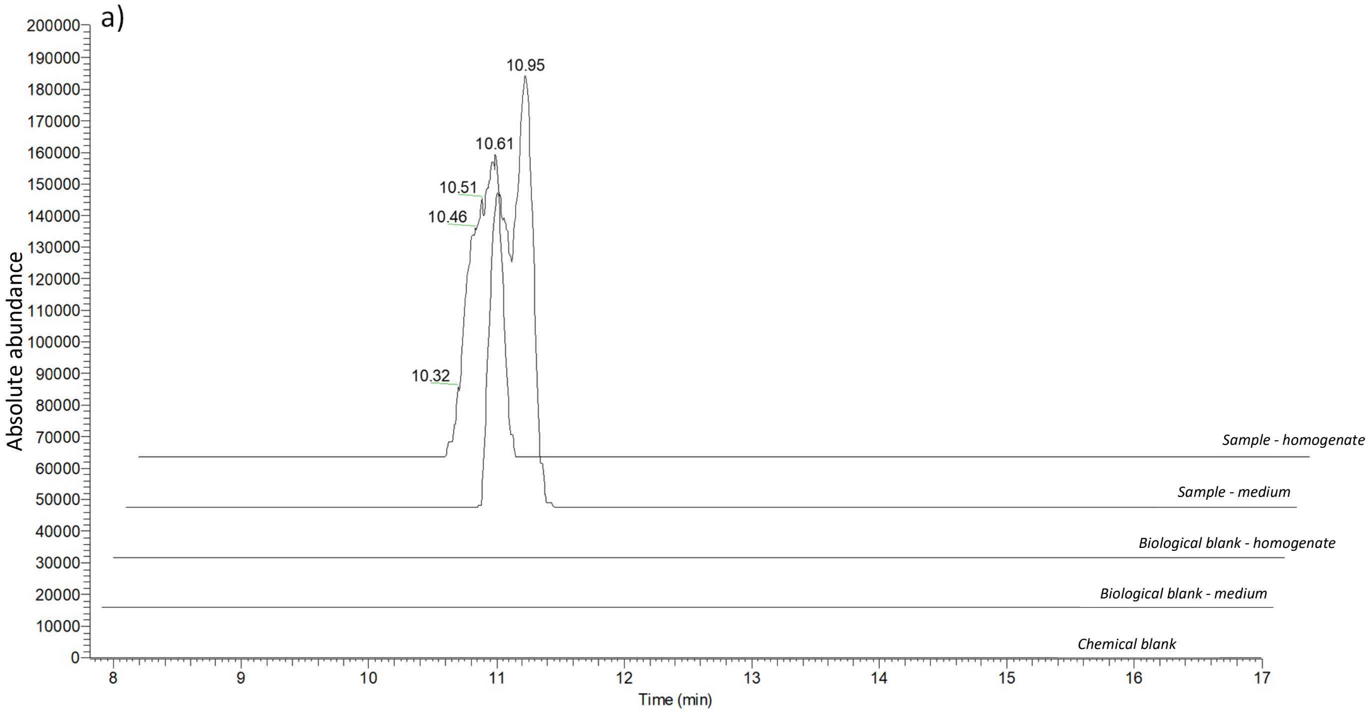

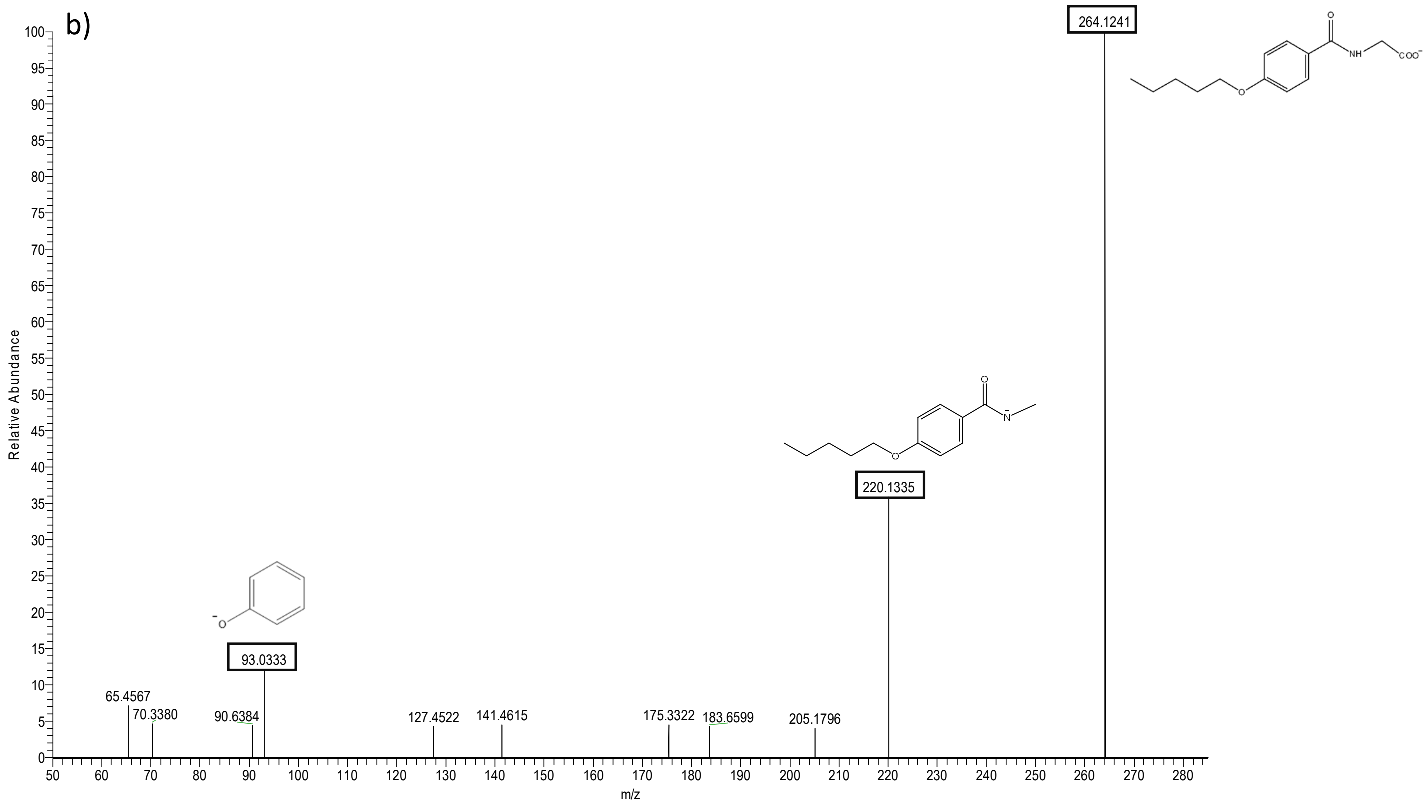

Figure S6: UHPLC chromatograms and HRMS/MS spectrum of h-BLK-gly. (a) Comparison of extracted ion UHPLC-HRMS chromatograms for *m/z* 264.1246 $[M\text{-}H]^-$ (**h-BLK-gly**) from isolated hepatocytes incubated with BLK127 (*sample–homogenate* and *sample–medium*), blank biological samples (*biological blank-homogenate* and *biological blank-medium)* and *chemical blank*. **(b)** HRMS/MS spectrum of BLK127-gly from the *sample–homogenate* with proposed structures of the main fragments.
