# Supplementary figures and images for: Assessing the Anthelmintic Candidates BLK127 and HBK4 for Their Efficacy on Haemonchus contortus Adults and Eggs, and Their Hepatotoxicity and Biotransformation
Source: Pharmaceutics. 2022 Mar 30;14(4):754. doi: 10.3390/pharmaceutics14040754 (PMC9024958; doi:10.3390/pharmaceutics14040754)

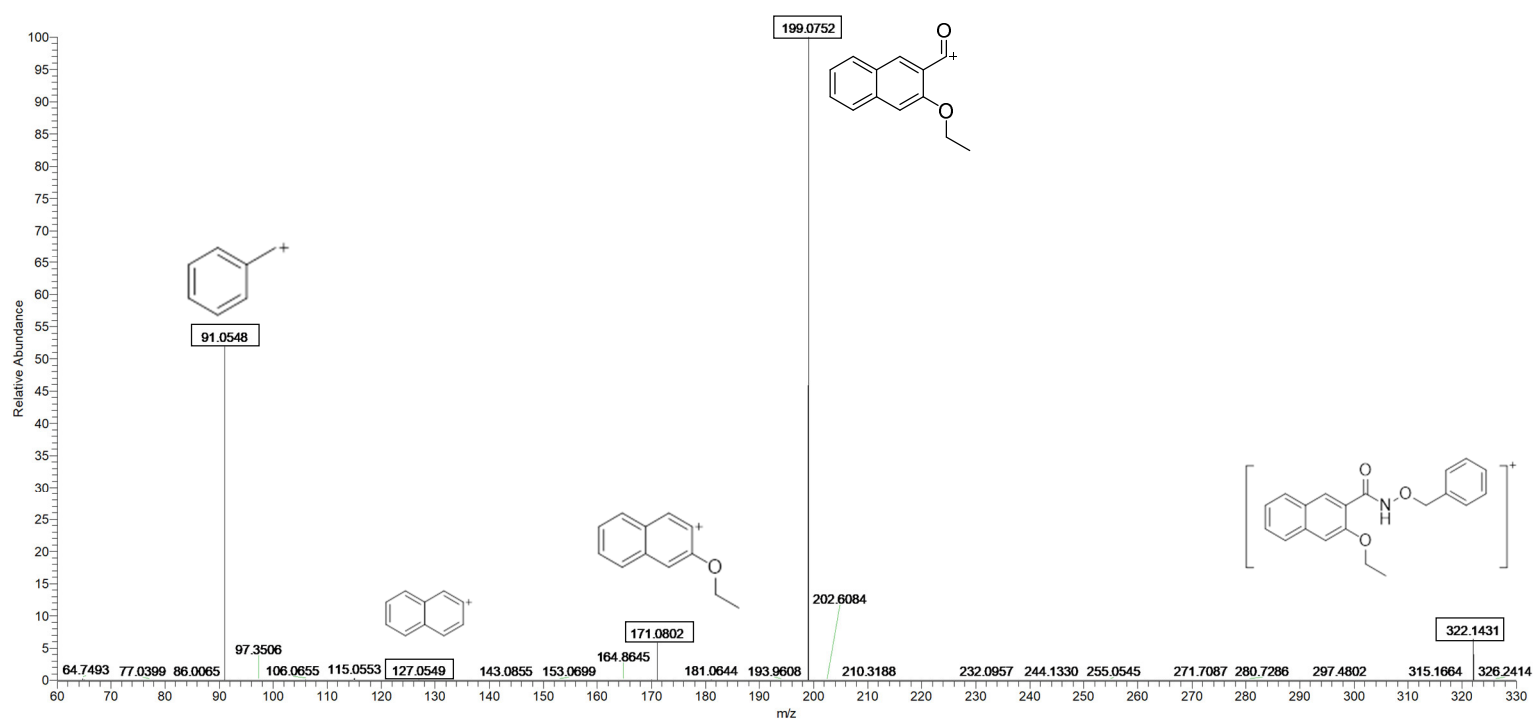

Figure S7: HRMS/MS spectrum of IS ( $m/z$  322.1431  $[M+H]^+$ ) from the standard.

Supplement: Supplementary file 1 [file pharmaceutics-14-00754-s001.zip › pharmaceutics-1649146-supplementary/Figure S7_HRMS-MS spectrum of IS from the standard.pdf]
